# *KIF21A* Gene c.2860C>T Mutation in CFEOM1A: The First Report from Iran

**Published:** 2018

**Authors:** Masoomeh Ramahi, Abolfazl Rad, Ebrahim Shirzadeh, Maryam Najafi

**Affiliations:** 1. Department of Biology, Sabzevar branch, Islamic Azad University, Sabzevar, Iran; 2. Cellular and Molecular Research Center, Sabzevar University of Medical Sciences, Sabzevar, Iran; 3. Department of Ophthalmology, Sabzevar University of Medical Sciences, Sabzevar, Iran; 4. Genome Research Division, Department of Human Genetics, Radboud University Medical Center, Geert Grooteplein Zuid 10, 6525 KL, Nijmegen, The Netherlands

**Keywords:** Fibrosis of extra ocular muscles, Iran, Mutation, Prenatal diagnosis

## Abstract

Congenital Fibrosis of the Extra Ocular Muscles1 (CFEOM1) is an autosomal dominant condition, caused by mutation in the *KIF21A* and *TUBB3*. It is characterized by congenital non-progressive restrictive ophthalmoplegia and ptosis. Mutational analysis of the known genes in such rare diseases by Sanger sequencing not only prevents wasting the time and expenses but also speeds diagnosis process, genetic counseling, and the possibility of prenatal diagnosis. Here, for the first time, association of pathogenic variant c.2860C>T in *KIF21A* gene in an Iranian family with positive history of CFEO-M1A was reported.

## Introduction

Congenital Fibrosis of the Extraocular Muscles (CFEOM) is characterized by congenital non-progressive ophthalmoplegia with or without ptosis affecting part or all of the oculomotor and/or the trochlear nucleus with its related nucleus and nerve 1. According to the clinical difference in the phenotype, CFEOM is subdivided to seven types including CFEOM1 (OMIM 135700) 1, CFEOM2 (OMIM 602078) 2, CFEOM3A (OMIM 600638) 3, CFEOM3B (OMIM 135700) 4, CF-EOM3C (OMIM 609384) 5, Tukel syndrome (OMIM 609428) 6, and CFEOM5 (OMIM 61004) 7. Literature reviews revealed pathogenic variants in the *TUBB1* (Tubulin Beta 1 Class VI), *TUBB2* (Tubulin Beta 2 Class II), *TUBB3* (Tubulin Beta 3 Class III) 3, *TUKLS* (Tukel syndrome) 6, *KlF21A* (Kinesin Family Member 21A) 8, *COL2A1* (Collagen Type XXV Alpha 1 Chain) 7 and *PHOX2A* (Paired Like Homeobox 2a) 2 genes in different types of CFEOM.

Classic CFEOM shows bilateral ophthalmoplegia with the eyes fixed in an infraducted position about 20 to 30 degrees below the horizontal midline. But CFE-OM3 phenotype has more variable clinical features as unilateral eye involvement and may be able to raise the eyes above midline 9. Inheritance pattern of CFEOM5, CFEOM2 and Tukel syndrome is autosomal dominant but CFEOM1 and CFEOM3 are autosomal recessive 10.

The first time, Yamada *et al* reported mutations in the *KIF21A* in 45 patients with CFEOM1 phenotype 8.

This study for the first time reported association of c.2860C>T *KIF21A* in the CFEOM1A phenotype in an Iranian family.

## Case Presentation

**Figure 1.**
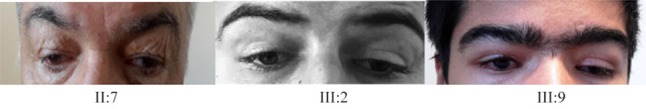
External photograph of II:7, III:2, III:9.

**Figure 2.**
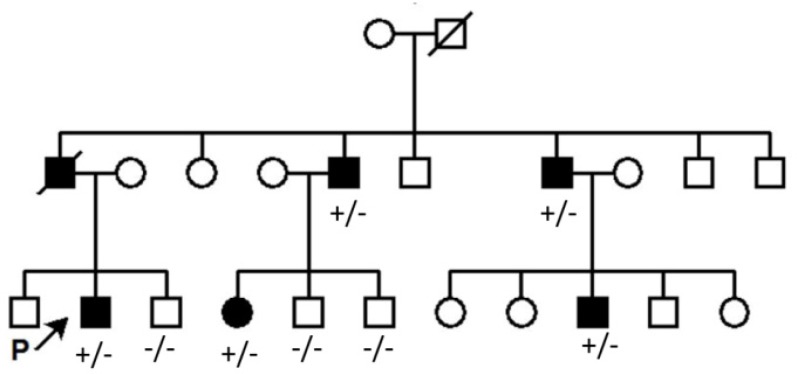
Pedigree related to the family with clinically affected CFEOM (1A). (+/−): Affected, (−/−): Wild type.

Proband was a 31-year-old man (III2) referred to Ophthalmology Department, Vasei Hospital on Dec. 2016 with severe bilateral restricted eye movements and ptosis since birth (Figure 1). His intellectual and social ability were satisfying and there were no other clinical symptoms as growth parameters abnormality, abdominal, respiratory and cardiovascular problems. Eye examination showed significant limitation of abduction, limitation of adduction and limitation of depression bilaterally. To compensate ptosis, 20 degree chin-up head position was noted. Fundoscopic observation detected no pigmentary retinopathy and optic atrophy. Pupillary function and anterior segment examinations were within normal limits. Due to the positive family history with similar ocular abnormalities across three generations (Figure 2), proband and his family received clinical genetic service.

Patient II:7 is a 54 year old man who was born with bilateral ophthalmoplegia and ptosis. Levator function was absent in both eyes. Primary vertical position of each eye was infraducted. Patient III:9 was a 14 year old boy who was born with typical signs of ptosis and complete restriction in eye movements. Ptosis was slightly improved after surgery at the age of 6 in the right eye.

All 3 patients had a normal cornea, iris, lens, and fundus appearance. Phenotype of the referring family has been suspected to be similar to the CFEOM 1. For time and cost saving, instead of doing Whole Exome Sequencing (WES) or performing Sanger sequencing on the known genes, according to the literature reviews, only *KIF21A* and *TUBB3* were sequenced which are involved in the most common form of CFEOM.

### Sanger sequencing

Ethical committee of Sabzevar University of Medical Sciences confirmed the study. Consent form was collected from all the members of the family that participated in the study. For performing molecular experiments, 5 *ml* peripheral blood was collected from each sample and was kept in EDTA tubes. According to the extraction kit (C.N. DN 8115C Sina colon, Iran), genomic DNA was extracted from peripheral blood. Considering the mutation reports of *KIF21A* and *TUBB3* in the literatures, exons 8, 20, 21 of the *KIF21A* gene and exons 1, 2, 3, 4 of *TUBB3* gene were amplified using sequence specific primers (Table 1). Optimal temperature conditions were as following: 5 *min* at 95*°C*, 35 cycles of 30 *s* at 95*°C*, 30 *s* at 57*°C*, and 1 *min* at 72*°C*. Then, Sanger sequencing was performed on purified amplicons (high throughput *Applied Biosystems 3730XL* sequencers). To analyze the results, the sequences were monitored using Finch TV software version 1.4.0.

**Table 1.** *TUBB3* and *KIF21A* primer sequences for conventional PCR

**Gene (exon/s)**	**Forward sequence**	**Reverse sequence**	**Amplicon size**
***tubulin beta-3(1)***	CAGCTCCTCTGGGAGACA	CATCCCTTTGTTGCAGGTT	485 *bp*
***tubulin beta-3(2)***	GAGGGCTAAAAGGCTTCACA	GGTGCTGAGACCTGGTCAGT	272 *bp*
***tubulin beta-3(3)***	CGGGCACAGAATTCAGAAA	TCATGTGAGGAGCTGACCAT	300 *bp*
***tubulin beta-3(4)***	TGCCCTTGGGATGTTCAG	GGGATCCACTCCACGAAGTA	846 *bp*
***tubulin beta-3(4)***	GTTCGATGCCAAGAACATGA	AGCTCTTCTTGCCTGTCCAC	862 *bp*
***KIF21A(8)***	TTTTAGCATTTTAGGTGCTTTT	AAAGTGCCAGCCTTAGATGT	306 *bp*
***KIF21A(20–21)***	TGTTGTACTTAAATGAAAAAATGGCTC	AGAGAAATCTGAAAAGCAAGCAGG	794 *bp*

## Results

**Figure 3.**
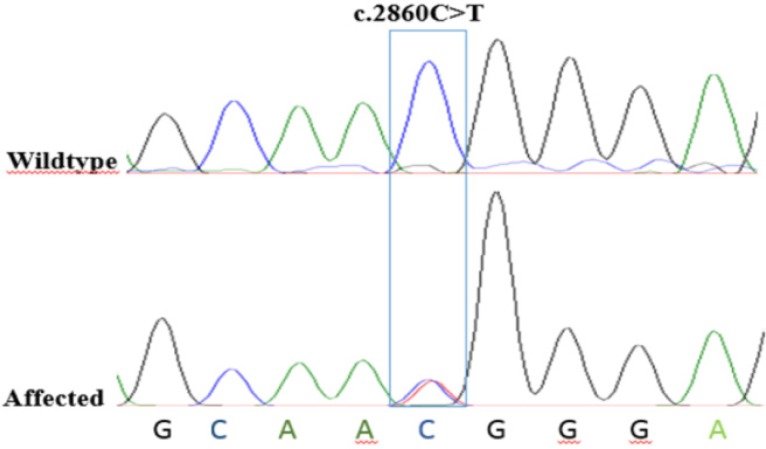
Chromatographs of Sanger sequencing. The healthy parents harbor normal DNA sequences, whereas the patient with CFEOM exhibits a c.*2860C*>*T* mutation at the second nucleotide position of codon 954 (p.Arg954Trp) on exon 21 of the *KIF21A* gene locus.

Data showed a heterozygote mutation c.2860C>T in the exon 21 of the *KIF21A*. c.2860C>T mutation changed the 954^th^ amino acid of *KIF21A* from Arginine to Tryptophan (p. Arg954Trp). For validating the pathogenic variant, segregation was extended on the rest of family members (wild type and patient individuals). Segregation results confirmed c.2860C>T variant in the patients (Figure 3).

## Discussion

In this paper, for the first time, the association of pathogenic variant c.2860C>T in *KIF21A* gene in an Iranian family with positive history of CFEOM1A was reported. NM_001173464.1 (*KIF21A*): c.2860C>T is known in ClinVar, uniport and dbSNP databases as a pathogenic variant and predictor tool such as phyloP, Grantham, SIFT and Mutation Taster if this change is deleterious and disease causing [Alamut Visual version 2.9 (Interactive Biosoftware, Rouen, France)].

CFEOM1 is subdivided to CFEOM1A and CFEO-M1B with mutation in *KIF21A* and *TUBB3*, respectively 11. CFEOM1A is the most common form of CFEO-M1 with autosomal dominant inheritance pattern that is characterized by congenital non-progressive restrictive ophthalmoplegia and ptosis 12.

*KIF21A* with 38 exons is located on 12q12 chromosome. It proceeds microtubule-stabilization through balancing polymerization and depolymerization 13. Mutational analysis of *KIF21A* in CFEOM1 confirmed 13 different missense mutations (c.84C>G, c.1056C->G, c.1067T>C, c.2830G>C, c.2839A>G, c.2840T>G, c.2840T>C, c.2841G>A, c.2860C>T, c.2861G>A, c.2861G>T, c.3022G>C, c.3029T>C) and a deletion c.3000_3002delTGA (p.Asp1001del) at codon 1001 14,15. Mutated *KIF21A* probably leads to CFEOM1 through failure in transferring cargo essential to the development of the oculomotor axons, neuromuscular junction or extraocular muscles 8. Our data was in line with the previous reports and existing in silico predictors.

**Figure 4.**
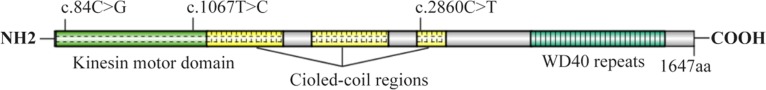
*KIF21A* mutations. Scheme of the Kinesin protein structure and the relative most common variant locations of *KIF21A*.

Chan *et al* genotyped a pedigree with CFEOM1 phenotype from Iran which they did not find any known variant 16. In figure 4, 3 of most common pathogenic variants are illustrated that disrupt the conserved regions of the *KIF21A* protein. Interestingly, in the present study, it was indicated that Iranian CFE-OM1A cases develop disease by the same way as cases from Japan, Hung Kung, America, and Europe. *KIF21A* is a causative gene in more than 50% of CFEOM1 phenotype in the case reports that were not hitherto pointed in Iranian families 14. Recurrence risk ratio of affected offspring is approximately 50% in each generation in CFEOM1.

## Conclusion

Therefore, results of this study demonstrated that genetic clinicians should refer to the literature reviews to record the last update of known genes in the rare disease prior to do Whole Exome/Genome Sequencing (WES/WGS) which most of families couldn’t afford it. Sanger sequencing of known genes not only saves time and needs less cost but also facilitates prognosis, genetic counseling and Prenatal Diagnosis (PND).
